# Smoking among Nurses in Turkey: Comparison with Other Countries

**Published:** 2007-03

**Authors:** Hafize Sezer, Nuran Guler, R. Erol Sezer

**Affiliations:** ^1^ Department of Biostatistics, Medical Faculty; ^2^ School of Nursing; ^3^ Department of Family Medicine, Medical Faculty, Cumhuriyet University, Sivas, Turkey

**Keywords:** Smoking, Nurses, Tobacco use, Turkey

## Abstract

The purpose of this study was to obtain baseline information on smoking among nurses. An attempt was made to contact, in person, all 301 nurses working for the university hospital in Sivas, Turkey, and when contacted they were asked to complete an anonymous questionnaire. Each unit of the hospital was visited three times, and 239 (79%) nurses were reached who all completed the questionnaire. Of the 239 respondents, 107 (45%) were current smokers, reflecting a substantially higher prevalence compared to that among the adult female population. The quit ratio was 22.5%. Of 127 ever-smoker nurses who responded to the related item, 90% started smoking during or after nursing education. This pattern of smoking initiation was different from the pattern in developed countries where nurses had already started smoking before beginning that training. Nurses with a high school education had a significantly higher prevalence of ever-smoking. Most respondents frequently or sometimes saw doctors smoking in rooms for nurses and in rooms for doctors in inpatient services. In-depth qualitative studies are needed to determine the reasons for the different smoking-initiation pattern.

## INTRODUCTION

The prevalence of smoking among nurses in Turkey is substantially higher than that among adult female population (1–6). Similar results were reported from Spain (7), Italy (8), and Japan (9) during the last two decades. Comparable results were also reported in the USA (10) and in the UK (11, 12) during the 1970s. The last two decades have witnessed a downward trend in the prevalence of smoking among nurses in many industrialized countries. Consequently, the reported rates of prevalence among nurses from these countries now remain at or below the prevalence of smoking among the adult female population (13, 14). The currently-reported decline in the prevalence of smoking among nurses has failed to keep pace with physicians and dentists in those countries. Although the prevalence of smoking among physicians in the USA, the UK, Australia, and Canada is ≤4% (14), the prevalence of smoking among nurses in these countries is 13–26% (15–19).

Because of their higher rates of smoking, nurses—especially those in countries like Turkey—are exposed to a higher risk of tobacco use-related morbidity and mortality. It is intuitive that a nurse can effectively coun-sel patients on cessation of smoking during clinical interactions (20) and that being a smoker can prevent a nurse from being active in smoking-cessation activities (15, 21). Therefore, we attempted to obtain baseline information on smoking, cessation of smoking, and related factors among nurses for the purpose of building a smoke-free nurses initiative. The period when nurses started to smoke was also focused on.

## MATERIALS AND METHODS

We attempted to contact, in person, all 301 nurses working for the Cumhuriyet University Hospital in Sivas, Turkey, to complete an anonymous questionnaire. The questionnaire was previously validated on 15 hospital nurses and was modified to improve the comprehensi-bility of the text. Confidentiality was assured through person-to-person communication. Each unit of the hospital was visited three times in the first three months of 2002, and 239 nurses (79%) were reached who all completed the questionnaire. The remaining 62 (21%) nurses were followed up by telephone and were asked two simple questions on the classification of their smoking status. These were the questions originally used in the National Health Interview Surveys of the US (23), and they were also included in our questionnaire survey. The questions were as follows: (a) Have you ever smoked 100 cigarettes in your entire life? and (b) Do you smoke cigarettes now?

The subjects responding ‘no’ to the first question were classified as non-smokers, while those answering ‘yes’ to the first question and ‘no’ to the second question were classified as former smokers. Those who responded ‘yes’ to both the questions were classified as current smokers. Ever-smokers included both former smokers and current smokers. Participants who responded—“yes, I smoke every day”—to the second question were defined as daily current smokers, while those who responded—“I don't smoke every day, but I smoke on some days”—to the second question were defined as some-day current smokers. To identify when they smoked most of their first 100 cigarettes, participants were asked two questions. In the questionnaire, age, marital status, type of nursing school, and presence of any other smokers in the household were the variables for which an association with smoking and/or quitting was sought. To describe the smoking situation in the hospital, nurses were asked how often they saw doctors smoking in some places (e.g. rooms for nurses, rooms for doctors in inpatient services) of the hospital.

Data were collected in 2002, and SPSS for Windows was used for obtaining results and for conducting statistical analysis. The chi-square test, the Cochrane test (24), one-way analysis of variance, and the Bonferroni test (for post-hoc comparisons) were conducted to determine statistical significance.

## RESULTS

Of the 239 nurses who participated in the questionnaire survey, 234 (98%) were women. Their mean age was 27.5 (standard deviation 5.3) years (median 26, range 20–53 years). Ninety-eight percent (n=234) were aged ≤40 years, 42% (n=100) were married, 57% (n=137) were single, and 1% (n=2) were divorced. There was no statistically significant difference regarding smoking status between the respondents of the questionnaire survey and the telephone survey. Five male nurses (two smokers and three never-smokers) were excluded from analysis.

Of the 234 female nurses who completed the questionnaire, 105 (45%) were current smokers, 31 (13%) were former smokers, and 98 (42%) were never-smokers. Of the current smokers, 23% (n=25) smoked ≤5 cigarettes per day, 33% (n=35) smoked 6–10 cigarettes per day, 24% (n=26) smoked 11–19 cigarettes per day, and 20% (n=21) smoked at least 20 cigarettes per day. No-one reported any other kinds (pipe, cigar, etc.) of tobacco-use.

The frequency distribution of ever-smokers by age at which they smoked most of their first 100 cigarettes is shown in the figure. Of the 127 ever-smoker nurses who responded to the related item, the percentage of those who smoked most of their first 100 cigarettes before, during, and after nursing education, was 10% (n=12), 40% (n=51), and 50% (n=64) respectively, and no-one reported initiation of smoking after the age of 28 years.

**Fig.**
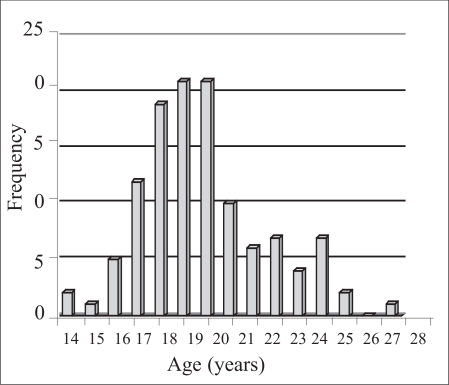
Frequency distribution of ever-smokers by age at which they smoked most of their first 100 cigarettes

The prevalence of ever-smoking indirectly standardized by age was 77.6% among respondents who had a high school education. It was 56.5% among those who graduated from a two-year nursing programme in a university and 50.9% among those who graduated from a four-year course (BSN) of nursing in a university. The differences between the former sub-group and any of the latter two sub-groups were statistically significant (p<0.05 and p<0.01 respectively) with the Cochrane test. The mean age at which ever-smokers smoked most of their first 100 cigarettes was 18.8 years for those nurses who had a high school education, 20 years for those who had graduated from a two-year programme in nursing in a university, and 20.5 years for those who graduated from a four-year course of nursing in a university (p=0.042 with one-way analysis of variance). Among post-hoc comparisons of the groups regarding mean age at which ever-smokers smoked most of their first 100 cigarettes, the only statistically significant difference was the one between the high school group and the four-year university group (p=0.04 with Bonferroni test). While the quit ratio was 54% among ever-smokers who did not have any other smokers in the household, it was 7% among those who had other smokers in the household (c^2^=37.9, p=0.00). Of the 106 nurse smokers who responded to the item relating to smoking at home, 46 (43%) smoked at home without any restriction. Of the 187 respondents who answered the question relating to their reaction to seeing someone smoking at the hospital despite ‘no smoking signs’, 97 (52%) ask them not to smoke. Of the 169 respondents who answered the question—“Do you inquire about your patients' smoking status during nursing services?”, 97 (57%) responded positively. Of the 225 nurses who answered the question—“Have you ever attended a course or a seminar or a lecture on smoking?”, 39 (17%) answered ‘yes’. Most respondents frequently or sometimes saw doctors smoking in rooms for nurses and in rooms for doctors in inpatient services.

## DISCUSSION

The observed prevalence (45%) of smoking among nurses in the Cumhuriyet University Hospital was much higher than those reported for the adult female population in Turkey which varied between 13% and 24% (4, 5). It was also higher than that among female physicians working in the same hospital which was reported to be 33% for 2000 (22). However, it appears to be similar to, and in the range of, the smoking-prevalence figures for nurses (40.3–68.6%) presented in a review by Tezcan and Yardim of 22 studies on smoking among health professionals in Turkey (6). Seven of these 22 studies used in this review were on smoking among nurses in six different provinces. Two other prevalence studies conducted during 1994–1999 in another province also reported smoking-prevalence figures among nurses in the same range given above (2, 3). The prevalence of smoking found in our study and other studies conducted in Turkey and cited above far exceeded the prevalence of smoking among nurses reported from Finland, the USA, the UK, Canada, and Australia which varied between 7% and 26% (15–19, 25) and was similar to those reported from Greece (26), Italy (8), and Spain (7). The observed quit rate (22.5%) in this study was substantially lower than the reported quit rates among nurses in developed countries; for example, the quit rate reported for nurses in Sydney was 51.7% (19).

The finding that only 10% of the ever-smokers reported initiation of smoking before nursing education in our study is different from the findings reported from industrialized countries. It was reported that 82% of nursing students in a London teaching hospital who smoked had been smoking on entry into nursing (27). McKenna *et al*. surveyed a sample of 2,000 qualified nurses in Northern Ireland and found that 96.8% of these nurses who smoked had taken up the habit prior to starting nursing (28). Wagner surveyed the smoking behaviour of 504 registered nurses in New York and found that the majority of them who smoked did so prior to starting their nursing careers (29). In their comprehensive review of literature from several industrialized countries, Rowe and Macleod Clark showed that the majority of nurses who smoked chose to do so before coming to the nursing profession (13). In summary, the majority of nurses who reported that they smoked in our sample started to smoke after coming to nursing, but the majority of nurses who smoked in industrialized countries started to smoke before coming to nursing.

Those nurses who had a high school education had a significantly higher prevalence of ever-smoking. The mean age when they began smoking was also significantly lower than that for nurses who graduated from a BSN programme. Two studies examined the educational level of nurses as a factor associated with smoking in industrialized countries and found no significant differences between the prevalence of smoking across groups of nurses who had different educational levels (28, 30).

The finding that the higher rate of quitting among ever-smokers who did not have any other smokers in the household than those who had suggests that the absence of any other smokers in the household might be an important predictor of successful long-term quitting among nurses. The absence of any other smokers in the household was reported to be a significant contributor for successful quitting in another study (31). Because of the high prevalence of smoking among adult males in Turkey, Turkish nurses who smoke appear to be under a negative factor at home with regard to quitting.

The pattern of smoking-initiation among nurses observed in this study (i.e. starting smoking during and after nursing education) was different from the pattern in developed countries where nurses started smoking before starting their nursing education. In-depth qualitative studies are needed to determine the reasons for this difference. The high rates of prevalence of smoking and the smoking-initiation pattern among Turkish nurses indicate an urgent need for interventions towards stopping smoking of nurses which should be started as early as possible in nursing education.
